# Pressure‐Induced Indirect‐Direct Bandgap Transition of CsPbBr_3_ Single Crystal and Its Effect on Photoluminescence Quantum Yield

**DOI:** 10.1002/advs.202201554

**Published:** 2022-08-10

**Authors:** Junbo Gong, Hongxia Zhong, Chan Gao, Jiali Peng, Xinxing Liu, Qianqian Lin, Guojia Fang, Shengjun Yuan, Zengming Zhang, Xudong Xiao

**Affiliations:** ^1^ School of Physics and Technology Wuhan University Wuhan 430072 P. R. China; ^2^ School of Mathematics and Physics China University of Geosciences (Wuhan) Wuhan 430074 P. R. China; ^3^ School of Physical Sciences University of Science and Technology of China Hefei Anhui 230026 P. R. China; ^4^ College of Mathematics and Physics Chengdu University of Technology Chengdu Sichuan 610059 P. R. China

**Keywords:** high pressure, indirect bandgap, lead halide perovskites, photoluminescence

## Abstract

Despite extensive study, the bandgap characteristics of lead halide perovskites are not well understood. Usually, these materials are considered as direct bandgap semiconductors, while their photoluminescence quantum yield (PLQY) is very low in the solid state or single crystal (SC) state. Some researchers have noted a weak indirect bandgap below the direct bandgap transition in these perovskites. Herein, application of pressure to a CsPbBr_3_ SC and first‐principles calculations reveal that the nature of the bandgap becomes more direct at a relatively low pressure due to decreased dynamic Rashba splitting. This effect results in a dramatic PLQY improvement, improved more than 90 times, which overturns the traditional concept that the PLQY of lead halide perovskite SC cannot exceed 10%. Application of higher pressure transformed the CsPbBr_3_ SC into a pure indirect bandgap phase, which can be maintained at near‐ambient pressure. It is thus proved that lead halide perovskites can induce a phase transition between direct and indirect bandgaps. In addition, distinct piezochromism is observed for a perovskite SC for the first time. This work provides a novel framework to understand the optoelectronic properties of these important materials.

## Graphical abstract

High pressure exerted on CsPbBr_3_ single crystal lead to the photoluminescence quantum yield dramatically improved more than 90 times due to the decreased dynamic Rashba splitting. Application of higher pressure transform it into a pure indirect bandgap phase, which can be maintained at near‐ambient pressure. These phenomena provide a novel framework to understand the optoelectronic properties of perovskite materials.

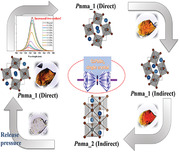

## 1 Introduction

Lead halide perovskites are of particular interest due to their numerous remarkable optical and electrical properties, which make them promising candidates for various optoelectronic applications.^[^ ^1^ ^]^ Notably, CsPbBr_3_, which has reasonable thermal stability, high electron mobility, a long carrier lifetime, and high photoluminescence quantum yield (PLQY), is attractive for solar cells, photodetectors and light‐emitting diodes (LEDs).^[^ ^2^ ^]^ In contrast to polycrystalline films and nanocrystals, grain boundary‐free single crystal (SC) perovskites, possessing higher carrier mobilities and longer diffusion lengths due to the dramatically lower density of trap states, may offer superior optoelectronic properties and the potential to boost device performance.^[^ ^3^ ^]^

Despite the common understanding that CsPbBr_3_ is a direct bandgap semiconductor with dipole‐allowed emission, it is extraordinary that bulk CsPbBr_3_ SC usually displays very low PLQY (typically ≈1% compared with ≈90% for its nanocrystals or quantum dots in solution). Due to the aggregation‐caused quenching (ACQ) effect,^[^ ^4^ ^]^ most lead halide perovskites, with or without organic ions, in the solid form of nanocrystals or quantum dots also suffer from low emission efficiency, which greatly impedes their applications in optoelectronic devices.^[^ ^5^ ^]^ Understanding the mechanism of low emission efficiency, and increasing it in solid lead halide perovskites, in particular bulk perovskite SCs, is critically important for applications such as LEDs.

To understand the low PLQY of CsPbBr_3_ SC, its energy band structure, and that of other metal halide perovskites, has been studied in detail. Wu et al. applied temperature‐dependent and time‐resolved spectroscopy to metal halide perovskite SCs containing organic or inorganic A‐site cations and confirmed the existence of indirect tail states below the direct transition edge.^[^ ^6^ ^]^ Time‐resolved photoconductivity measurements have revealed that excitation below the direct gap into an indirect band leads to the photogeneration of mobile carriers, which exhibit slower recombination than carriers generated by excitation of the direct band.^[^ ^7^ ^]^ These new findings suggest that the fundamental bandgap may be indirect in these halide perovskite systems.^[^ ^8^ ^]^ The indirect band is only slightly shifted in *k*‐space (*k_0_*), and is predicted to be at a slightly lower energy than the direct band. This could have an enormous impact on the dynamics of charge carriers: light absorption may proceed through direct transitions, but recombination of cooled carriers may be slowed by the band extrema shift in *k*‐space, leading to a forbidden indirect transition.^[^ ^8^, ^9^ ^]^ Notably, Wu et al. suggested that the presence of indirect tail states caused by Rashba splitting implies that it is difficult for any lead halide perovskite SC to function as lasing media, considering that they show no amplification of spontaneous emission at room temperature, or even at low temperature.^[^ ^6^ ^]^ However, these suggestions have not drawn widespread attention because this Rashba effect is not readily exploitable in applications. Furthermore, it remains controversial as to what extent the crystal structure affects the photophysics of lead halide perovskites. From a physics perspective, the structure–function relationship in differently sized perovskites remains poorly understood. In addition, the indirect bandgap of these perovskite materials is extremely small, which has caused most researchers to ignore its existence. The question remains: is it possible to obtain pure‐indirect‐bandgap lead halide perovskites?

Application of external pressure can be used to precisely manipulate crystal and band structures, and tune the electronic parameters of crystals.^[^ ^10^ ^]^ Although high‐pressure studies of metal halide perovskites, including CsPbBr_3_, have been reported by several groups,^[^ ^11^ ^]^ almost all of the experiments were performed on powdered samples, nanocrystals, or quantum dots, which are not ideal for accurate structural determinations of highly pseudosymmetric materials, nor for spectroscopic studies (the interaction of grain boundaries can influence optical characterisation) and microscopic observations. Particularly, the photoluminescence (PL) of perovskites is typically significantly deteriorated under high‐pressure conditions because of loss of quantum confinement by agglomeration.^[11c,12]^ For the same reason, i.e., the agglomeration of grain boundaries, previous high‐pressure studies conducted on lead halide perovskites generally showed amorphization under high pressure (usually above 2–5 GPa), which limited exploration of the intrinsic properties of perovskite materials under high pressure.^[^ ^11^ ^]^ Based on the above considerations, we carried out high‐pressure experiments using a symmetric diamond anvil cell (DAC) on a high‐quality CsPbBr_3_ SC, for which grain boundaries could be ruled out. The optical properties, including absorption and PL spectra, were systematically studied and supported by microscopic observations.

## 2 Results and Discussion

### 2.1 Absorption Spectra and Photoluminescence Spectra Under Pressure

**Figure 1:**
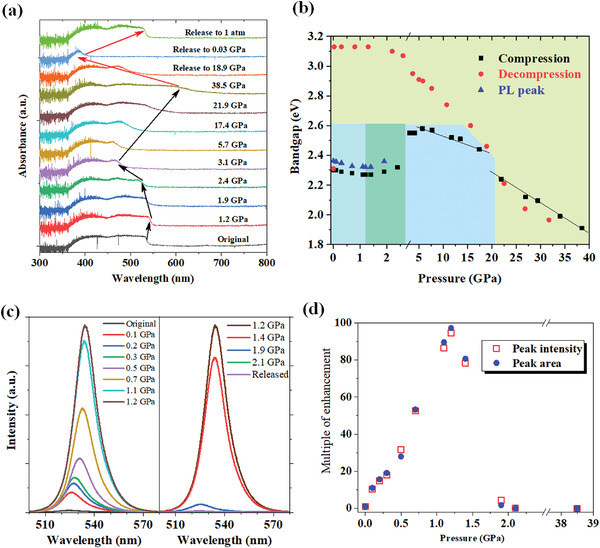
a) Absorption spectra of CsPbBr_3_ SC under applied pressure. b) Bandgap energy is determined from the absorption onset measured in the compression (black) and decompression (red) runs. Blue triangles show the PL peak position during the compression run. c) PL spectra of CsPbBr_3_ SC under applied pressure. d) Enhancement factor of PL for the peak intensity (red open squares) and for the integrated peak area (blue filled circles), both with respect to PL under ambient pressure.

The absorption and PL spectra of CsPbBr_3_ SC were obtained under hydrostatic pressure to study the evolution of pressure‐induced optical properties. The sample was the same as that previously used,^[^ ^13^ ^]^ and its X‐ray diffraction pattern was consistent with the expected pure orthorhombic (*P*nma) symmetry. Selected steady‐state absorption and PL spectra are shown in **Figure** **1**a,c, respectively. Under ambient conditions, a steep absorption onset edge located at 539.1 nm and weak green PL centered at 525.0 nm were observed. The sharp absorption edge indicated that the CsPbBr_3_ SC was likely a direct bandgap material, consistent with previous literature reports.^[^ ^14^ ^]^ With increasing pressure, both the absorption edge and PL peak position gradually red‐shifted up to 1.2 GPa, and then gradually blue‐shifted from 1.4 to 2.4 GPa. Most surprisingly, the PL intensity exhibited unambiguous and significant pressure‐sensitive evolution in this pressure range. As the pressure increased from ambient to 1.2 GPa, the corresponding PL intensity showed a remarkable enhancement of about two orders of magnitude, which is markedly different from previous results for nanocrystalline and powdered samples.^[11c,g]^ Considering that the PLQY of our CsPbBr_3_ SC at ambient condition was determined to be ≈1.0%, the PLQY of this sample under 1.2 GPa of pressure should have been nearly unity. Admittedly, to conduct a high‐pressure experiment, the sample was only micrometer‐scale which was placed in the center of stainless‐steel gasket. (Figure S1, Supporting Information) It is unrealistic to obtain the PLQY value of the sample directly under high pressure. Even though, this is the first time that CsPbBr_3_ bulk materials with high PLQY have been reported. As the pressure continued to increase above 1.2 GPa, the PL intensity rapidly decreased and almost disappeared above 2.4 GPa.

Increasing the pressure from 2.4 to 3.1 GPa caused a sudden blue‐shift of the absorption edge. Notably, the absorption edge changed from the previously sharp onset to a gradual one, which implied a transition from a direct to indirect bandgap. The abrupt change in absorption edge and disappearance of PL indicated that the electronic states of the SC sample changed at this pressure, likely due to a structural phase transition. Further increasing the pressure from 3.1 to 17.4 GPa caused the absorption edge to red‐shift continuously but slowly. At still higher pressure, the absorption edge red‐shifted relatively more quickly.

The above changes in absorption spectra were quantitatively assessed in terms of bandgap energy by extrapolating the edge position. The bandgap evolution during compression (Figure 1b) clearly indicated four pressure regimes: 0–1.2 GPa, where the bandgap slowly red‐shifted (2.30 → 2.27 eV) and PL intensity dramatically increased; 1.4–2.4 GPa, where the bandgap slowly blue‐shifted (2.27 → 2.32 eV) and PL intensity rapidly decreased until completely disappearing; 3.1–17.4 GPa, where the bandgap underwent a sudden blue‐shift relative to the previous stage and displayed an abruptly flattened absorption edge, followed by a continuous red‐shift (2.55 → 2.44 eV); and 17.4–38.5 GPa, where the bandgap red‐shifted relatively more quickly (2.44 → 1.91 eV). These four pressure regimes indicated four different phases of CsPbBr_3_ SC under compression.

Upon pressure release, the absorption and PL did not revert to their states in the compression run. The SC sample's bandgap monotonically increased with decreasing pressure until the pressure approached 1 atm. Notably, when the pressure decreased to 0.03 GPa, the bandgap of CsPbBr_3_ remained at an unexpectedly large 3.13 eV, which is much higher than any value observed during the compression process. The SC sample suddenly recovered to the initial 2.30 eV when the pressure returned to 1 atm (0.0001 GPa). Meanwhile, the characteristics of the absorption edge also underwent a transition from indirect to direct bandgap, which is the same as the initial state before pressurization. The absorption spectrum of CsPbBr_3_ SC recovered to its original state under ambient conditions, while its PL spectrum reappeared with somewhat lower intensity (Figure 1c). This suggests that the crystal structure was reversible. However, the high‐pressure phase did not recover until 0.03 GPa was reached, which is very close to ambient conditions; this suggests an irreversible process.

To explore the structure change of CsPbBr_3_ SC after pressurization, the X‐ray diffraction pattern of single crystal sample before pressurization, and after being released to ambient pressure for 10 min have been collected as shown in Figure S2, Supporting Information. It was found that there was no significant difference between them. The identical XRD patterns of them combined with the reversible absorption spectrum shown in Figure 1a indicated that the phase transition of CsPbBr_3_ under high pressure was an entirely reversible process.

We conducted a second compression run to examine the repeatability of the pressure‐induced changes. The evolution of the absorption spectra was consistent with the first run, while the overall PL intensity was clearly lower than that of the first run (Figure S3, Supporting Information). Previous researches indicated that the PL intensity of CsPbBr_3_ can be reduced under laser irradiation due to the light related surface defects.^[^ ^15^ ^]^ Our own experiment also proved that the PL intensity can decreased 70% after continuous UV light illumination ≈20 min. (Figure S4, Supporting Information) Consider that during the whole compression process, PL spectra were collected many times under 325 nm excitation. It is understandable for the PL intensity decline during the second run of measurement.

**Figure 2:**
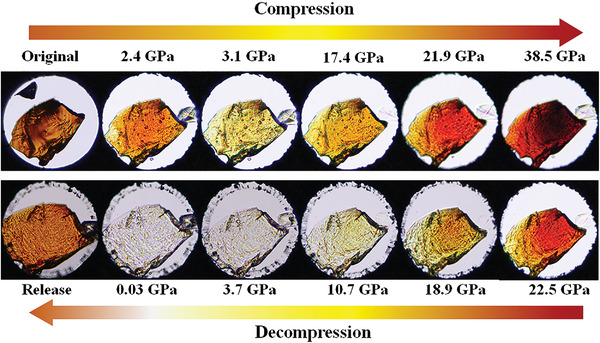
Optical microscopy of CsPbBr_3_ SC during a compression and decompression run.

Optical micrographs of CsPbBr_3_ SC under various pressures were obtained to visually demonstrate the phase transition process (**Figure** **2**). We clearly observed for the first time the piezochromism of CsPbBr_3_ SC corresponding to the evolution of the optical properties of the crystal. The CsPbBr_3_ SC was initially orange at ambient pressure, but turned yellow at ≈3.1 GPa and red at ≈21.9 GPa. During decompression, the color changed first to light yellow and was then colorless and transparent after the pressure reached ≈3.7 GPa. This colorless state persisted until 0.03 GPa. Once the pressure had completely released to ambient, the color of the sample returned to orange within a few minutes. This color change process is consistent with the evolution of the bandgap (Figure 1b).

Previously, perovskite single crystals usually were considered as possess low PLQY and were difficult to function as LEDs or lasing media at room temperature. In this work, we proved that high‐PLQY perovskite SC can be obtained under high pressure and open the door for perovskite SC to be applied as LEDs or lasing media. Although low‐cost LED has been widely used and it is uneconomical for a high‐pressure material to be used as LED. However, the high‐PLQY characteristic under high pressure make it became possible for perovskite SC used as lasing media once amplified spontaneous emission at room temperature was realized. Besides, the remarkable pressure‐induced bandgap change and piezochromism phenomena of CsPbBr_3_ SC can be used as a visual pressure sensor.

### 2.2 Theoretical Calculations and Correlations with the Experiments

**Figure 3:**
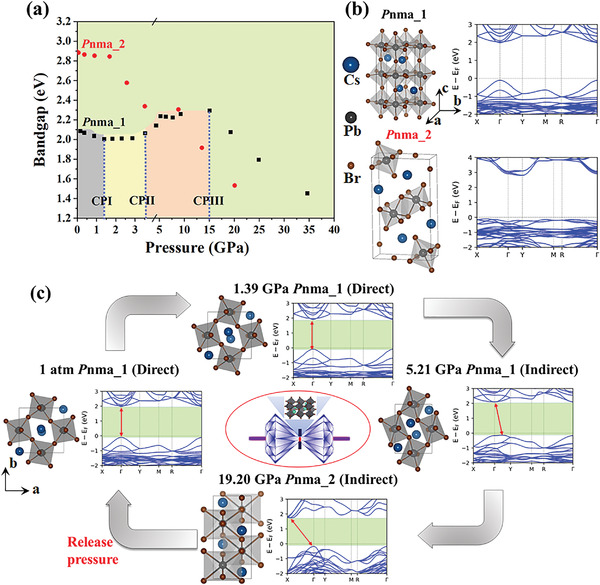
a) Calculated bandgaps of two orthorhombic CsPbBr_3_ phases as a function of pressure. Black and red dots are respectively for the initial structures of *P*nma_1 and *P*nma_2. b) The atomic and band structures of *P*nma_1 and *P*nma_2. c) Evolution of atomic and electronic structures of CsPbBr_3_ SC under four representative pressures.

We performed first‐principles calculations of the structural and electronic properties of CsPbBr_3_ SC as a function of applied pressure, to better understand the evolution of its electronic structure. High‐throughput structural searches identified two structures of the *P*nma orthogonal space group, namely *P*nma_1 and *P*nma_2, having similar total energies under ambient conditions. The total energy of *P*nma_1 is ≈70 meV lower than that of *P*nma_2 (Figure S5, Supporting Information), indicating that *P*nma_1 is slightly more stable than *P*nma_2 and thus represents the structure of CsPbBr_3_ SC at ambient pressure. The atomic and energy band structures of these two phases are shown in **Figure** **3**b,c, respectively. Although the structures have the same symmetry, their lattice constants are markedly different. Specifically, the *P*nma_1 structure has nearly identical lattice constants in the *ab*‐plane, with a *b*/*a* ratio of 1.03, while the *P*nma_2 structure possesses very different lattice constants in the *ab*‐plane, with a *b*/*a* ratio of 2.20. This difference leads to very different electronic structures (Figure 3). For example, the *P*nma_1 structure has a direct band gap of 2.089 eV at the Γ point, while the *P*nma_2 structure shows a much larger indirect band gap of 2.885 eV. Notably, the former is widely accepted both theoretically and experimentally for CsPbBr_3_ at ambient conditions, whereas there has been no experimental report of the latter to date.

The atomic and band structural properties of *P*nma_1 under hydrostatic pressure were studied based on the lowest total energy structure calculations. Figure 3(a) presents the bandgap value as a function of compression. Three critical pressures were identified, i.e., CP‐I at ≈1.4 GPa, CP‐II at ≈4.0 GPa, and CP‐III at ≈15 GPa. Below CP‐I, CsPbBr_3_ maintained the *P*nma_1 structure (direct bandgap) and underwent bandgap reduction from 2.089 to 2.008 eV with increasing pressure. Once the pressure exceeded CP‐I, an isostructural phase transition occurred. Although the structure remained *P*nma_1 with direct bandgap, the bandgap increased from 2.008 to 2.144 eV with increasing pressure. At pressures above CP‐II, the crystal structure still displayed *P*nma_1 characteristic, while the bandgap became indirect and displayed a slightly increasing value. The calculations indicated two coexisting structures as the pressure increased from CP‐III, i.e., *P*2_1_/m (direct bandgap) and *P*nma_2 (indirect bandgap). Considering that the experimental absorption spectra of CsPbBr_3_ above 20 GPa had an indirect bandgap with a clival absorption tail, it is likely that the CsPbBr_3_ underwent a structural phase transition to *P*nma_2 (indirect bandgap) at this point. This assignment is also supported by the lower total energy of *P*nma_2 compared with *P*2_1_/m (Figure S5, Supporting Information).

Based on our calculations and experimental results, the evolution of the geometric and electronic structures of *P*nma CsPbBr_3_ under the four critical pressures are summarized in Figure 3c. Although the CsPbBr_3_ SC remained in the orthorhombic phase (*P*nma) before and after CP‐II, a phase transition from direct to indirect bandgap with more severe octahedral rotation/distortion and lower structural symmetry occurred. When the applied pressure was below 15.04 GPa, the lattice constant *a* decreased quickly, while the lattice constant *b* increased slightly (Figure S6, Supporting Information), leading to the disappearance of isostructural lattice constants in the *ab*‐plane. Accordingly, the *b*/*a* ratio increased from 1.03 at the ambient condition to 1.73 at 15.04 GPa, which is close to the value of 2.20 in the *P*nma_2 phase having the anisotropic *ab* plane. Furthermore, when the pressure surpassed 15.04 GPa, the lattice constants *a*, *b*, and *c* decreased slightly (Figure S6, Supporting Information). Therefore, the direct‐to‐indirect and final indirect‐to‐direct transitions might be closely related to reduction and enhancement of crystal symmetry, respectively. Structural deviation or departure from high symmetry of perovskite crystals can change the space group, and the structural alteration can manifest in three ways, i.e., as octahedral tilt (rotation), cation displacement, and octahedral distortion. Figure 3 indicates that all three of these structural alternation components participated in the pressure‐induced structural distortion. In a relatively low‐pressure region below CP‐I, the main component was octahedral tilt, whereas in the pressure region above CP‐I, octahedral distortion started to play an important role.

Compared with those of the *P*nma_1 phase, the bandgap and lattice structure of *P*nma_2 under compression varied quite differently. With increasing pressure, the indirect bandgap feature remained unchanged, and the gap decreased monotonically. It seems that no structural phase transition occurred in this pressure region, since the *b/a* ratio was always at the initial value of ∼2.20 (Figure S7, Supporting Information). Notably, the *P*nma_2 phase is more stable than the *P*nma_1 phase under high pressure due to the lower total energy (Figure S5, Supporting Information). Thus, the structural phase transition from *P*nma_1 to *P*nma_2 under high pressure can be understood. Furthermore, this indirect bandgap phase remained during the decompression process until the pressure reached the near‐ambient condition. The proposed direct/indirect bandgap feature and values are consistent with the experimental observations. The sudden transformation from *P*nma_2 to *P*nma_1 during the decompression process under near‐ambient conditions is also explained. In summary, the theoretical results were in agreement with the experimental results. Notably, we judged that *P*nma_2 phase was obtained under high pressure based on the following considerations: 1. According to first‐principle calculations, the total energy of *P*nma_1 is only ≈70 meV lower than that of *P*nma_2 (Figure S5, Supporting Information), agreed with the experimental phenomenon that a sudden phase transformation during the decompression process under near‐ambient conditions occurred. 2. The bandgap difference between the experiment (from 3.1 eV to 2.3 eV) and calculation (from 2.885 eV to 2.089 eV) were both 0.8 eV. 3. The absorption spectra characteristic changed from gradual tail to sharp edge is also agreed well with the characteristic from indirect *P*nma_2 to direct *P*nma_1. 4. Experimentally, a small change in release pressure (0.03 GPa‐1 atm) cause a large change in bandgap (3.13 eV‐2.30 eV). This is exactly agreed well with the calculations. The small total energy difference (70 meV) indicated that *P*nma_1 was slightly more stable than *P*nma_2 while the situation will be immediately reversed if pressure increased a little (Figure S5, Supporting Information).

**Figure 4:**
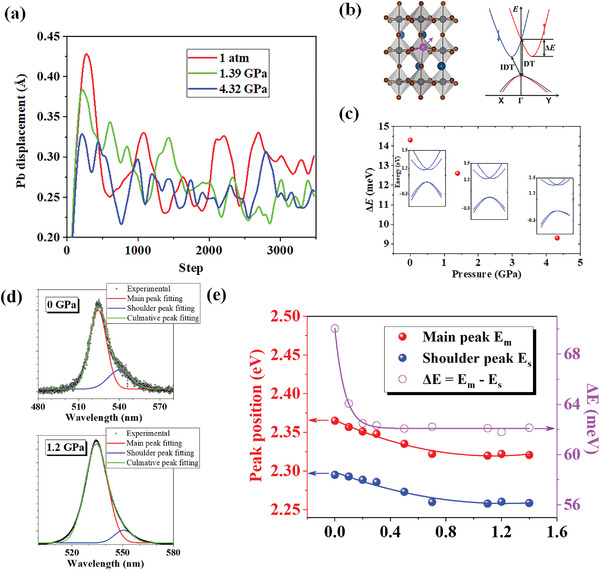
a) The Pb atom displacement in *P*nma_1 CsPbBr_3_ SC at room temperature under different pressure. b) Schematics of Pb atom displacement and the corresponding Rashba band structure. c) Rashba splitting of *P*nma_1 CsPbBr_3_ SC with the Pb atom displacement under three representative pressures. The insets show the corresponding Rashba electronic structures. d) Dual peaks Gaussian fitting for PL peaks under 0 and 1.2 GPa. e) Dual emission energy and the energy difference of PL peaks as a function of pressure.

The highly unusual enhancement of PL under pressure remains to be explained. Why is the PLQY of CsPbBr_3_ SC so low under ambient conditions as a direct bandgap material, and why did the PL intensity greatly increase after applying a pressure of ≈1.4 GPa? An indirect bandgap structure induced by Rashba splitting of the conduction band is often introduced to account for the low emission efficiency in organic‐inorganic hybrid lead halide perovskites.^[^ ^7^, ^16^ ^]^ The required broken centrosymmetry derives either from the structured organic cation or the distorted lead halide octahedron. While still under debate, a dynamic Rashba effect with nonharmonic thermal disorder could also provide the broken inversion symmetry and occur in pure inorganic lead halide perovskites. Bulk Rashba splitting is expected to occur when the inversion symmetry of a system is broken. There are two ways to break the inversion symmetry: statically and dynamically. Based on calculations, the lattice structure with the lowest total energy is unfortunately centrosymmetric. However, local structural fluctuations are intrinsic to lead halide perovskite crystals.^[^ ^17^ ^]^ With respect to the skeleton, the Pb atoms in orthorhombic CsPbBr_3_ may be displaced from their equilibrium position. **Figure** **4**a shows that under ambient conditions, the root‐mean‐square displacement (RMSD) of the Pb atom ranges from 0.23 to 0.42 Å, as calculated by molecular dynamics simulation, causing dynamic Rashba splitting. Moreover, as the pressure increases, the RMSD value of Pb atoms decreases.

The most probable polar Pb distortion (Figure S8, Supporting Information), i.e., ≈0.29 Å under ambient pressure, 0.26 Å under 1.39 GPa, and 0.25 Å under 4.32 GPa along the <111> direction, was used to quantify the dynamic Rashba splitting effect under various applied pressures. The typical Rashba band structure is presented in Figure 4b, where the conduction band minima for both spins slightly move away from the Γ point, while the valence band maxima remain located at the Γ point. Figure 4c shows the wave vector along which the conduction band minima move away from the Γ point by as much as 0.041Å^–1^ for the three chosen pressures. The Rashba splitting energy (*ΔE*) is 14.3, 12.6, and 9.3 meV, respectively, consistent with decreasing displacement resulting in decreasing *ΔE*.^[^ ^18^ ^]^ As the applied pressure increases from ambient to CP‐II, the *ΔE* decreases from 14.3 to 9.3 meV, indicating that the direct transition is enhanced, in turn enhancing the PL intensity. Our experimentally collected PL spectra confirmed the predicted change in *ΔE*. By applying multiple‐peak Gaussian fitting to the PL spectra range from 480 to 580 nm (Figure 4d), dual PL emission peaks were observed, which corresponded to direct and indirect bandgap emissions, respectively.^[^ ^6^ ^]^ This fitting method has been adopted by several groups to obtain direct and indirect characteristics of perovskite materials at ambient conditions. For example, Zhang et al. propose an exciton recombination process involving both indirect and direct transitions simultaneously according to the dual‐spectral feature of PL emission and long tail in absorption spectra at ambient condition.^[^ ^19^ ^]^ Broadly similar characteristics of multiple PL emission were also observed in this work. Different from previous work which conducted at ambient pressure, our experiment displayed the evolution of dual PL emission at high pressure. Figure 4e shows that when the pressure increased to 1.4 GPa, the main peak was red‐shifted by 44 meV while the shoulder peak was red‐shifted by 36 meV. Meanwhile, the difference in energy between the two types of emission decreased from ≈70 to ≈62 meV, thereby providing experimental evidence for the decreased Rashba splitting effect. The calculated dynamical Rashba splitting is apparently smaller than experimental result, which has also been reported by Wu et al.^[^ ^6^ ^]^ They attribute this difference to the imperfect lattice, polaron formation as well as the approximations in the calculation methods. Nonetheless, these theoretical and experimental reports lend crucial support for the dynamical Rashba effect.

Besides the effect of Rashba splitting, the dipole transition matrix element *P^2^* reveals the transition probabilities between the topmost valence and the lowest conduction band that contribute to the PL intensity.^[^ ^20^ ^]^ Considering that the PL intensity of the indirect transition related to phonon interaction is usually very low, only the *P^2^* around the direct transition Γ point was calculated. We summarized the *P* ^2^ of *P*nma_1 CsPbBr_3_ SC with Pb displacement at room temperature under different pressure in Table S1, Supporting Information. It is shown that the value of *P* ^2^ is always around 385 when the pressure was lower than CP‐I, while it decreased significantly from 385 at CP‐I to 220 at CP‐II, thus reducing by nearly half. Therefore, when the pressure ranges from CP‐I to CP‐II, the decreased dipole transition matrix elements dominated the PL intensity, resulting in the PL reduction. As a whole, at a pressure lower than CP‐I, the PL intensity dominated by dynamic Rashba splitting was dramatically enhanced by compression. When the pressure increased from CP‐I to CP‐II, the decreased dipole transition matrix elements were the dominant factor in the PL intensity. Summarizing, the PL intensity was first enhanced by pressure, reaching the maximum value at CP‐I, and then decreased with increasing pressure. The variation of direct/indirect transition constituents and dipole transition matrix led to the change of PL intensity. Notably, the phonon effects are not included in the dipole transition calculation, since the PL intensity of indirect transition related to phonon interaction is usually very low,^[^ ^20a^ ^]^ we thus only calculate the transition dipole moment around the direct transition Γ point. Actually, in the experiment, as can be seen from the normalized PL spectra shown in Figure S9, Supporting Information, all the PL spectra showed similar full width at half maximum (FWHM), which indicated that electron‐phonon interactions could hardly be influenced by pressure in this occasion.

Based on the finding of this work, we try to offer a possible explanation for the reason that CsPbBr_3_ and other lead halide perovskites frequently display a much higher PLQY as nanocrystals or quantum dots than in the bulk state. According to the Laplace equation, the internal pressure (*p*) in a spherical particle can be expressed as *2γ/r*, where *γ* and *r* are the surface energy of the material and radius of the particle, respectively.^[^ ^21^ ^]^ Thus, there is significant internal pressure in nanocrystals or quantum dots.^[^ ^22^ ^]^ Using a general surface energy value of 1 N/m for oxides, the internal pressure in fine particles having an average particle diameter of 20 nm is ≈200 MPa. Thus, the difference in PLQY between bulk and nano‐sized perovskites under ambient external pressure is explained by different internal pressures, which could be a novel supplement to the traditional quantum confinement effect theory.

## 3 Conclusion

We studied the optoelectronic changes of CsPbBr_3_ SC under high pressure (up to 38.5 GPa). The modification and evolution of the optical properties as a function of high pressure were explored in situ using optical absorption and PL spectroscopies. The bandgap changed remarkably with pressure, and the PLQY increased dramatically for more than 90 times at 1.2 GPa. This completely overturns the traditional concept that the PLQY of lead halide perovskite SC cannot exceed 10%. First‐principles calculations indicated that this abnormal PL enhancement was due to an increasing influence of the direct bandgap characteristic caused by decreasing dynamic Rashba splitting. At ≈3 GPa, CsPbBr_3_ SC underwent a phase transition to a pure indirect bandgap phase; this is the first experimental report of pure indirect bandgap CsPbBr_3_. This pressure‐induced indirect phase was maintained under near‐ambient conditions during the decompression process and recovered to the direct phase abruptly only when the pressure was completely released to ambient conditions. Our investigation proves that high‐pressure technology is an effective and practical approach to tune the optical properties and structure of lead halide perovskites.

## 4 Experimental Section

### Sample Preparation

The CsPbBr_3_ perovskite SC was prepared via a modified inverse‐temperature crystallization in water solution which has been elaborated in detail in the previous publication.^[^ ^13^ ^]^ CsBr and PbBr_2_ (molar ratio of 1:1) were dissolved in HBr solution (48 wt% H_2_O) to form saturated perovskite precursor solution. To guarantee full dissolution, the solution was stirred at 100 °C for 1 h. Then, the solution was filtrated and transferred to a clean container with a seed crystal placed on the bottom, which was placed on a stable hot plate and gradually cooled at a rate of 2 °C h^−1^ to room temperature. The crystal growth process took ≈36 h until large single crystals formed. The X‐ray diffraction pattern of the sample at ambient condition was also introduced in the previous publication.^[^ ^13^ ^]^ Powder X‐ray diffraction measurements were carried out on a XtaLAB PRO diffractometer at 1.542Å using Cu X‐ray generated by MMF007 rotating‐anode X‐ray (Rigaku, Japan) with Pilatus 200K detector in Core Facility Center for Life Sciences, University of Science and Technology of China. The data was processed and reduced using CrysAlisPro.

### High‐Pressure Experiment

A diamond anvil cell (DAC) was employed to provide a high‐pressure environment for CsPbBr_3_. The DAC was used and consists of a cylinder and a piston, which are pulled together with screws. Through tightening the four groups of screws on the cubic boron nitride seats, pressure was applied on diamond anvil cells and further compressed the sample. The well‐known pressure shift of the ruby luminescence of *R*_1_ line was used for pressure calibration.^[^ ^23^ ^]^ Such extreme pressures can be accurately determined by monitoring ruby fluorescence lines using the following relationship:

(1) $$P=\frac{1904}{B}\left[{\left(1+\frac{\Delta \lambda }{694.24}\right)}^{B}-1\right]$$

where *P* is the pressure in GPa, Δ*λ* is the ruby R1 line shift in nm, and parameter B is 7.665 forquasi‐hydrostatic conditions. CsPbBr_3_ crystal was packed into a hole with a diameter of 150 µm in a stainless‐steel gasket. Silicone oil was used as Pressure transmitting medium to generate a hydrostatic pressure environment for absorption and photoluminescence measurements. In situ Fluorescence spectra were obtained under reflection geometry at room temperature, using an integrated Raman spectrometer (LabRAM HR800 Jobin Yvon) equipped with a confocal microscope, a stigmatic spectrometer, and a multichannel air‐cooled CCD detector. 325 nm laser was utilized to collect the emission spectra of CsPbBr_3_ crystal. Absorption spectra under high pressure were collected by a home‐built confocal microscope system equipped with a photomultiplier tube detector (Zolix‐HVC1800), an ultraviolet‐visible spectrometer (Zolix‐Omni‐*λ*750), and a standard light source (EQ‐99CAL LDLS).

### Calculation Method

These calculations were performed using the projector augmented wave (PAW) method implemented in the Vienna ab initio simulation package code.^[^ ^24^ ^]^ The standard PAW pseudo potentials are adopted.^[^ ^25^ ^]^ The Perdew, Burke, and Ernzerhof (PBE) form of the generalized gradient approximation (GGA) exchange‐correlation functional for crystal structure relaxation and electronic structures was used.^[^ ^26^ ^]^ The cut‐off energy is set to 500 eV after convergence tests. A 3 × 3 × 2, 6 × 6 × 5, and 9 × 9 × 7 Γ‐centered Monkhorst–Pack k‐point grid for relaxations, self‐consistent calculations, and electronic structure calculations were employed, respectively. In the current calculations, the total energy is converged to less than 10–5 eV. The maximum force is less than 0.02 eV/Å during the optimization. The spin–orbit coupling (SOC) effects have been included for the electronic band structures for checking the Rashba splitting. Here, the volume of the structure was fixed, and relax the lattice constants and atomic positions fully to model the pressure. The pressure value was estimated by the relation *P* = ‐∂E/∂V, where *E* is the total energy of the structure and *V* is the fixed cell volume. This method has been verified to be effective to model pressure.^[^ ^27^ ^]^

## Conflict of Interest

The authors declare no conflict of interest.

## Supporting information

Supporting Information

## Data Availability

The data that support the findings of this study are available from the corresponding author upon reasonable request.
