# Supplementary material for: Pressure‐Induced Indirect‐Direct Bandgap Transition of CsPbBr3 Single Crystal and Its Effect on Photoluminescence Quantum Yield
Source: Adv Sci (Weinh). 2022 Aug 10;9(29):2201554. doi: 10.1002/advs.202201554 (PMC9561783; doi:10.1002/advs.202201554)
Supplement: Supplementary file 1 — Supporting Information [file ADVS-9-2201554-s001.pdf]

ADVANCED SCIENCE
Open Access

Supporting Information

for *Adv. Sci.*, DOI 10.1002/advs.202201554

Pressure-Induced Indirect-Direct Bandgap Transition of $CsPbBr_3$ Single Crystal and Its Effect on Photoluminescence Quantum Yield

Junbo Gong, Hongxia Zhong, Chan Gao, Jiali Peng, Xinxing Liu, Qianqian Lin, Guojia Fang, Shengjun Yuan, Zengming Zhang* and Xudong Xiao**

Supporting Information

Pressure-induced indirect-direct bandgap transition of $CsPbBr_3$ single crystal and its effect on photoluminescence quantum yield

Junbo Gong, Hongxia Zhong, Chan Gao, Jiali Peng, Xinxing Liu, Qianqian Lin, Guojia Fang, Shengjun Yuan, Zengming Zhang*, Xudong Xiao**

Dr. J. B. Gong, Zhong, Dr. J. L. Peng, Mr. X. X. Liu, Prof. Q. Q. Lin, Prof. G. J. Fang, Prof. S. J. Yuan, Prof. X. D. Xiao
School of Physics and Technology, Wuhan University, Wuhan, 430072, China
E-mail: gongjunbo@whu.edu.cn; xdxiao@whu.edu.cn

Dr. H. X.
School of Mathematics and Physics, China University of Geosciences (Wuhan), Wuhan 430074, China

Dr. C. Gao, Prof. Z. M. Zhang
School of Physical Sciences, University of Science and Technology of China, Hefei, Anhui 230026, China
Email: zzm@ustc.edu.cn

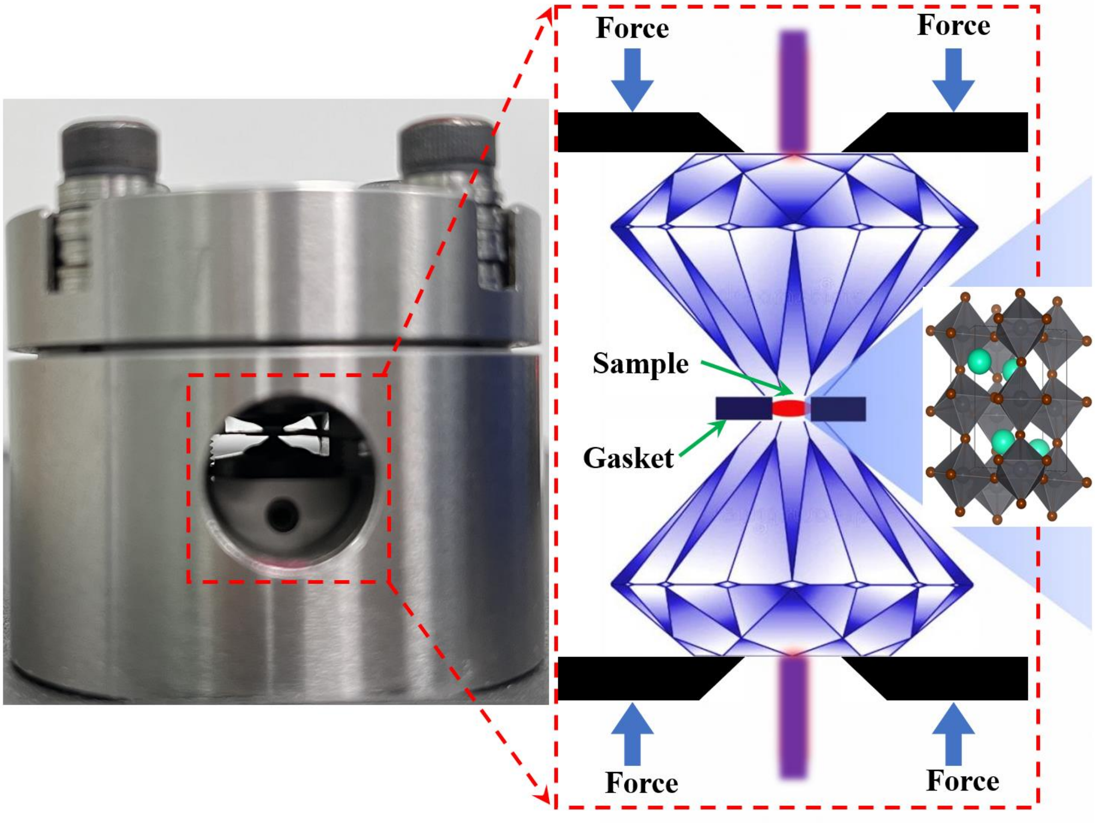

Figure S1 Photo and schematics of the used symmetric diamond anvil cell.

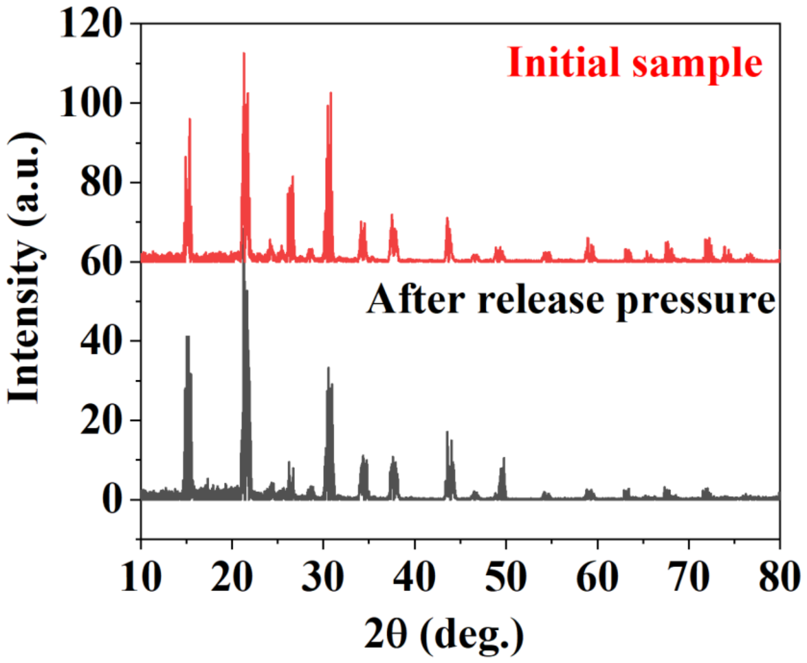

Figure S2 X-ray diffraction pattern of single crystal sample before pressurization, and after released to ambient pressure for 10 min.

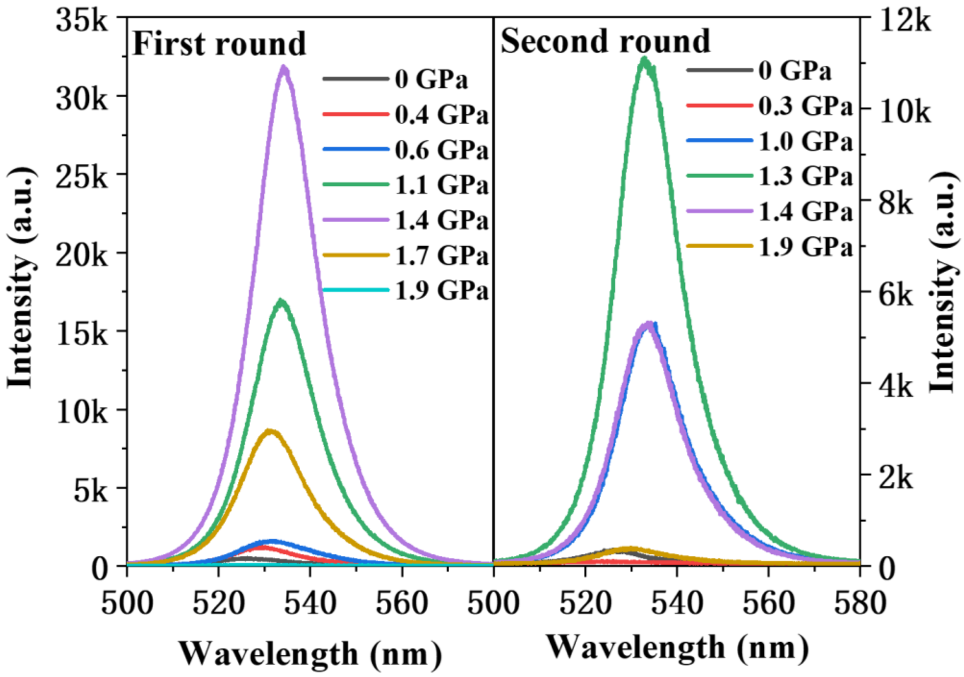

Figure S3 PL spectra at first run and second run.

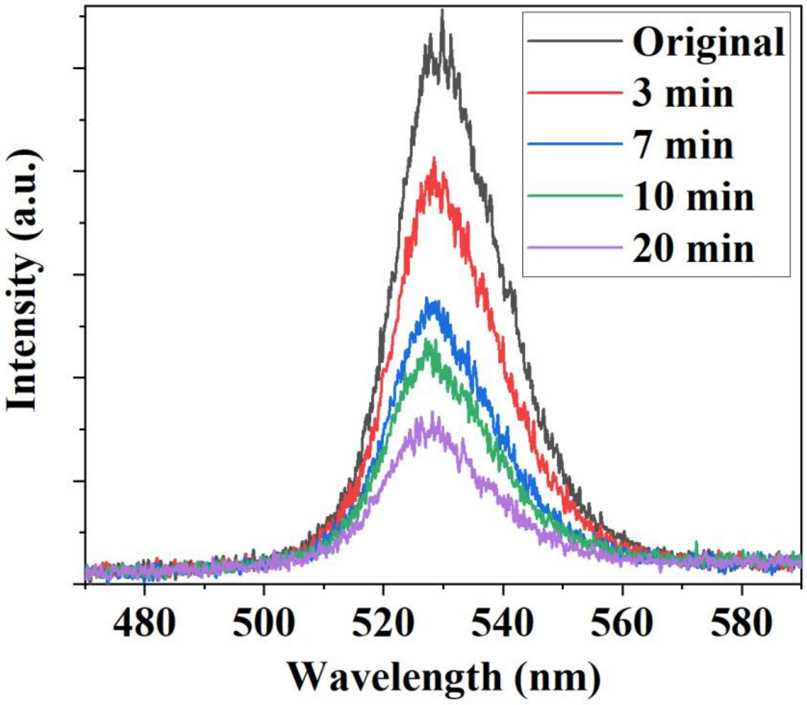

Figure S4 PL spectra of $CsPbBr_3$ SC after varied deposed time under UV light illumination under 0 GPa in the DAC.

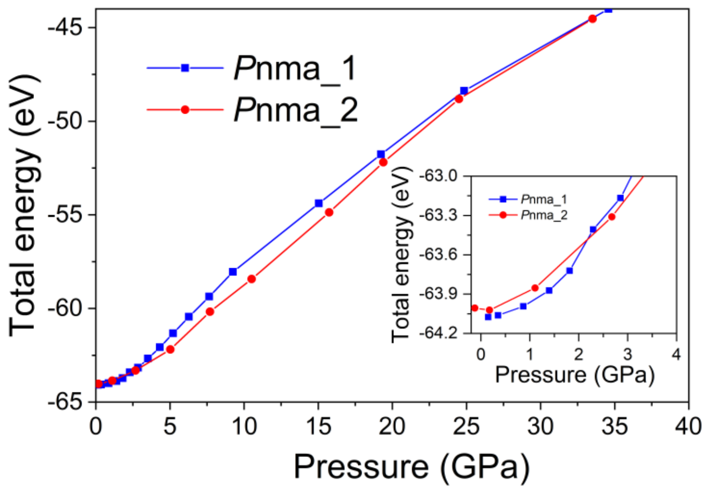

Figure S5 Calculated total energies of two orthorhombic $CsPbBr_3$ as functions of pressure. The inset zoom in the low pressure region. The blue (red) dots show the data of the initial structures *P*nma_1 (*P*nma_2). Near the ambient condition, the total energy of *P*nma_1 is lower than *P*nma_2, indicating the existence of *P*nma_1 at ambient condition. When the pressure keeps up going 15.04 GPa, the *P*nma_2 has lower total energy compared with that of *P*nma_1. In high pressure region, the *P*nma_2 is the stbale phase, which is observed in experiments.

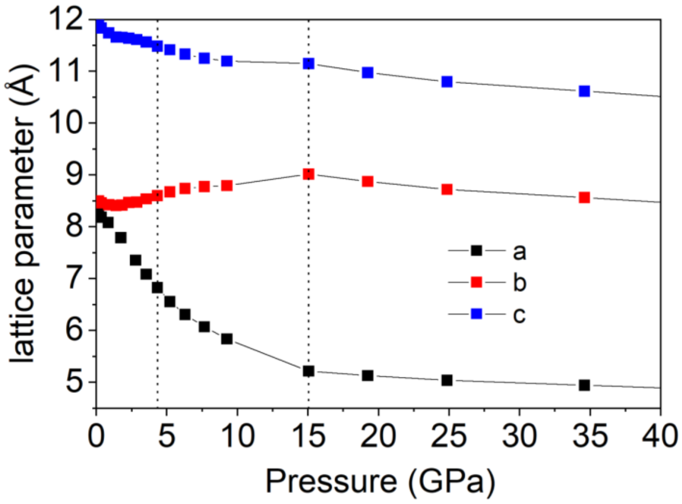

Figure S6 Cell parameter evolutions of $CsPbBr_3$ SC *P*nma_1 as functions of pressure. When the pressure is below 15.04 GPa, the lattice contant a decreases quickly, while lattice constant b increases. When the pressure is higher 15.04 GPa, the lattice constants a, b, and c decreases slightly.

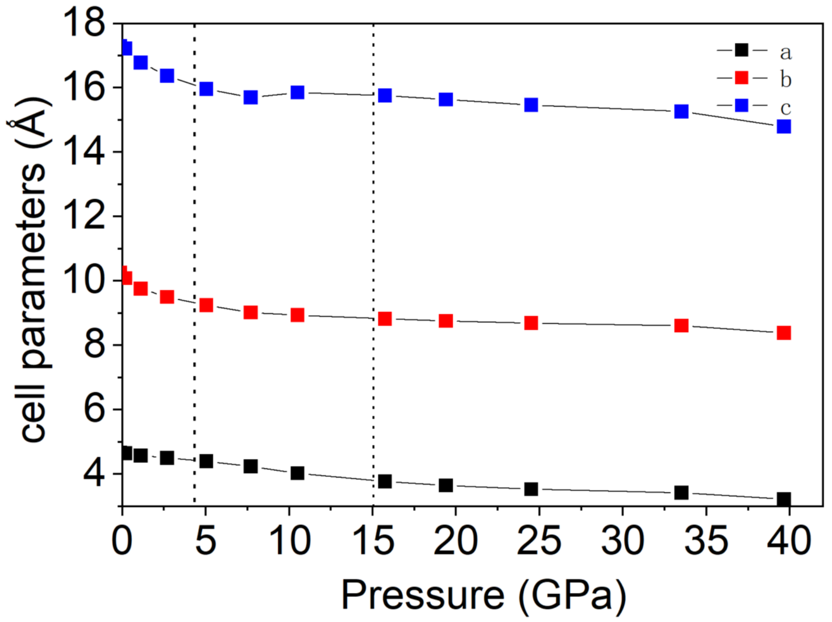

Figure S7 Cell parameter evolutions of $CsPbBr_3$ SC *P*nma_2 as functions of pressure. When the pressure is higher than 4.33 GPa, the lattice contants decreases slightly, whose trend is consistent with the $CsPbBr_3$ SC *P*nma_1 in this region.

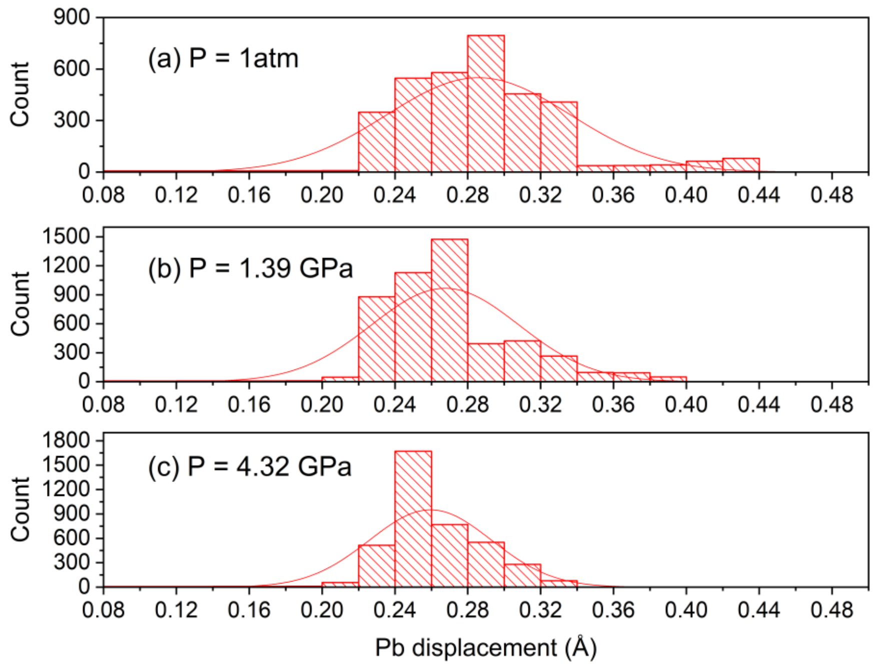

Figure S8 The Gaussian distribution of Pb displacement in $CsPbBr_3$ crystals *P*nma_1 under the considered three pressure.

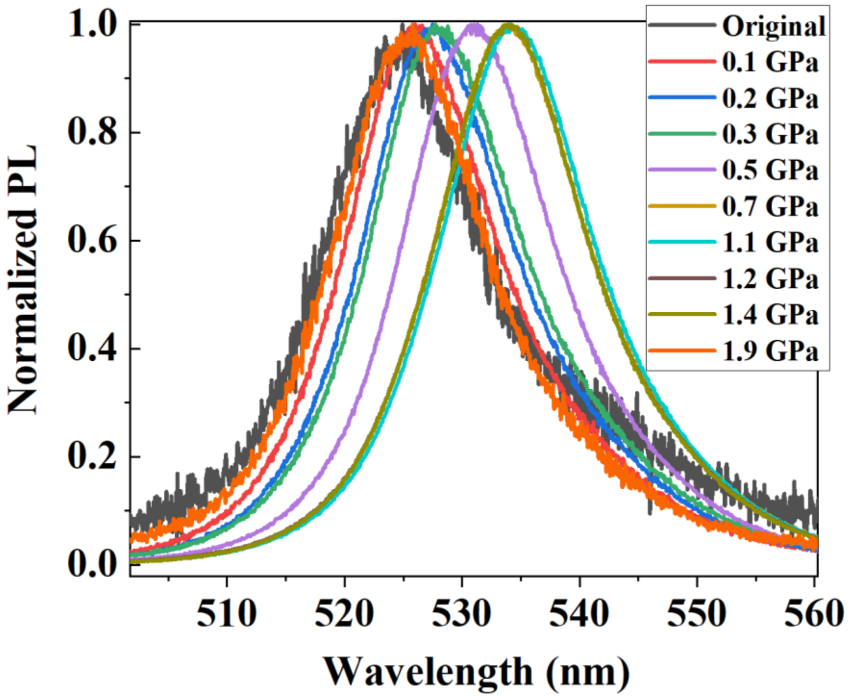

Figure S9 Normalized PL spectra of CsPbBr3 SC under applied pressure.

Table S1 Transition dipole moment P^2 of *P*nma_1 $CsPbBr_3$ SC with Pb displacement at room temperature under different pressure.

Pressure (GPa)	0	0.86	1.40	2.07	4.00
P^2 (a.u.)	385	400	392	340	220
